# Tetralogy of Fallot With Right Aortic Arch and Retro-Aortic Innominate Vein

**DOI:** 10.3389/fped.2018.00331

**Published:** 2018-11-02

**Authors:** Zhia N. Lim, Bryan J. W. Chew, Sanfui Yong, Antonio F. Corno

**Affiliations:** ^1^East Midlands Congenital Heart Center, Glenfield Hospital, Leicester, United Kingdom; ^2^Cardiovascular Research Center, University of Leicester, Leicester, United Kingdom

**Keywords:** congenital heart surgery, congenital heart disease, tetralogy of fallot, right aortic arch, vascular ring, retro-aortic innominate vein

## Abstract

Right aortic arch (RAA) and retro-aortic innominate vein are rare vascular anomalies. Diagnosis of these anatomical variations can be achieved using fetal echocardiography, post-natal echocardiography, and computed tomography scan. RAA can form a vascular ring when associated with other vascular anomalies which may compress the trachea and/or esophagus. On the other hand, the existence of retro-aortic innominate vein can influence the clinical decision-making and surgical strategy. We report a rare occurrence of both RAA and retro-aortic innominate vein in a 3 months old girl with a prenatal diagnosis of tetralogy of Fallot and include details of her presentation and successful management.

## Introduction

Right aortic arch (RAA) and retro-aortic innominate vein are both rare vascular anomalies, generally associated with other congenital heart defects (CHD), predominantly tetralogy of Fallot (1–4). When isolated, RAA does not usually produce symptoms in patients. However, when co-existing with other vascular anomalies, it may result in respiratory symptoms owing to compression of the trachea (5, 6). The retro-aortic innominate vein also does not cause symptoms on itself but has significance when performing cardiac surgery for palliation or repair of CHD (2).

## Case report

A 3 months old girl with a background of antenatally diagnosed CHD presented to outpatient department clinic with central cyanosis and significant inconsolable agitation. On examination, no murmurs were audible and oxygen saturation was shown to be between 48 and 66%. Immediate management was initiated with knee-to-chest positioning, intravenous fluid and morphine administration. This episode lasted for about 15 min.

The history revealed that this infant was born to a pair of non-consanguineous afro-Caribbean parents and was diagnosed antenatally in the second trimester with tetralogy of Fallot, followed by intrauterine growth restriction in the third trimester. Her birth was unremarkable, with vaginal delivery at term, weight 2.45 kg and APGAR scores of 9. Her genetic analysis showed a normal karyotype with no evidence of 22q11 deletion. Her baseline oxygen saturation was around 95% on room air and she had a grade 4/6 ejection systolic murmur.

After hospital admission for her first cyanotic spell, she was commenced on oral beta blockers and her oxygen saturations stabilized between 85 and 90% on room air, without any further hypoxic spells during her stay. She was then discharged home. Subsequently, she was re-admitted to hospital with recurrence of cyanosis and she suffered from multiple cyanotic spells daily over the course of this second hospital stay. These spells increased in frequency and length proportional to the length her stay; the longest spell lasting for more than 30 min. Despite medical management with posture adjustment, morphine use, fluid boluses and incremental increase in beta blockade (up to 2 mg/kg/dose), she did not show any improvement. In a span of a week, her baseline oxygen saturation had dropped from 85 to 70%; and oxygen saturation during a cyanotic spell was as low as 40%.

**Figure 1:**
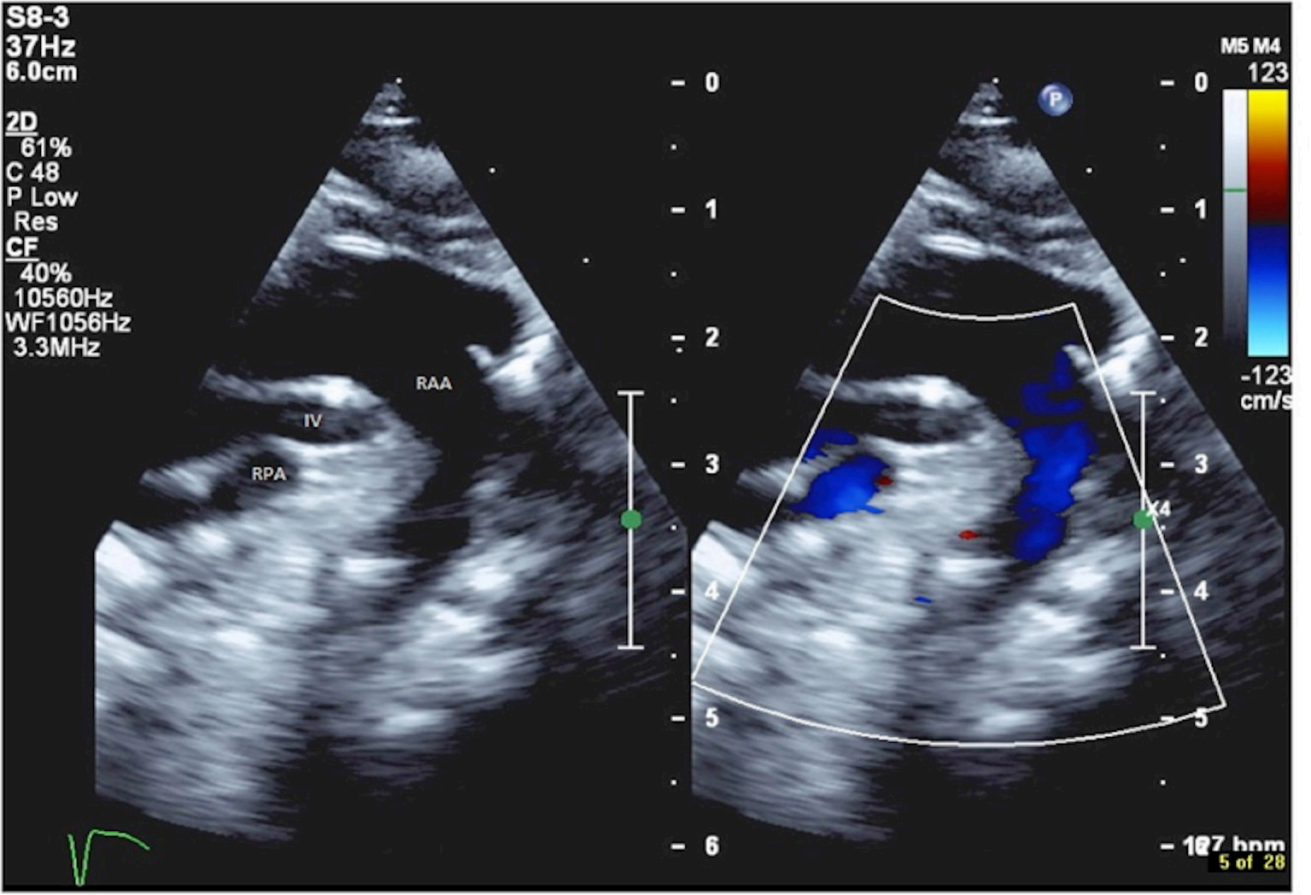
Echocardiography showing the retro-aortic position of the innominate vein (IV), positioned between the right aortic arch (RAA) and the right pulmonary artery (RPA).

Echocardiography confirmed the pre-natal diagnosis showing the morphology of tetralogy of Fallot with hypoplastic main pulmonary artery, right aortic arch, retro-aortic innominate vein, single right superior vena cava, and a normal coronary arteries pattern (Figure 1). Surgical repair of tetralogy of Fallot was planned during this hospital admission, at an age of 4 months and body weight of 4.67 kg. Operation was performed through median sternotomy, with normothermic cardio-pulmonary bypass, and consisted of patch closure of the ventricular septal defect and relief of the right ventricular outflow tract obstruction with resection of the infundibular obstruction and transannular patch extended to the main pulmonary artery.

The post-operative course was uneventful. Pre-discharge echocardiography showed good cardiac function and no residual intra-cardiac defects. Her post-operative oxygen saturation was 97% on room air and she remains clinically stable in good condition 2 months after hospital discharge.

## Discussion

We present a case of a 3 months old girl with tetralogy of Fallot associated with RAA and a retro-aortic innominate vein.

RAA is a rare vascular malformation where the aortic arch crosses over the right bronchus instead of the left. It occurs in 0.1% of pregnancies and accompanies other CHD, most commonly with tetralogy of Fallot (5, 7). The second vascular anomaly is the retro-aortic innominate vein, which arises from abnormal development of the cardinal venous system. This vascular malformation is incredibly rare, with an incidence of 5 in 1,000 of children with CHD and 2 in 10,000 children without CHD. This anomalous vein typically courses anterior to the trachea and posterior to the ascending aorta, under the aortic arch to join the right brachiocephalic vein before entering the superior vena cava (1–4, 6–8).

Detection of RAA prenatally with fetal echocardiography is crucial due to associated cardiac abnormalities, particularly trunco-conal malformations such as the tetralogy of Fallot, and chromosomal defects, notably 22q11 deletion. Furthermore, RAA, when associated with other vascular malformations can form a vascular ring with other anomalous vessels, with a strong potential to compress on the trachea and esophagus leading to symptoms such as stridor, wheeze and dysphagia (5, 7). Pre-operatively, our patient was experiencing frequent episodes of respiratory distress in the form of cyanotic spells as well as a general reduction in oxygen saturations on room air when settled. This occurred despite the optimization of medical management and supportive therapy. Blood tests, analysis of nasopharyngeal aspirate and plain chest radiograph ruled out infection as a potential cause for her presentation. The association of RAA and retro-aortic innominate vein in this 3 months old infant was most probably contributing to the respiratory complications requiring intensive peri-operative respiratory physiotherapy. Previous authors have reported that CHD and anomalies of the aortic arch and great vessels can cause airway obstruction and respiratory difficulties via several mechanisms (6, 7). Enlargement of the heart and great arteries which may occur in CHD can lead to external compression of the large airways which are smaller, softer and more pliable in infants than in adults. Furthermore, increased pulmonary artery pressure, either through an increase in pulmonary blood flow or a reduction in left sided cardiac output, may cause edema around the small airways and inevitably reduces pulmonary compliance (7). Respiratory distress in infants is associated with higher morbidity and mortality rates (7). Hence, clinicians should be aware of associated vascular anomalies in the context of a patient with CHD because of the high risk of potential obstruction of airways, in order to organize appropriate pre-operative and post-operative management.

In addition to being an anatomical rarity, with 0.2–1.0% incidence reported in the literature (1–4) and 1.9% in the Middle East population (4), the retro-aortic innominate vein has some clinical significance. It is important to be aware of the presence of this anomalous vein, as difficulty could arise during transvenous pacemaker insertion or central venous line placement if the procedure was carried out via the left-arm approach. Also, it is important for the retro-aortic innominate vein to be known pre-operatively to the cardiothoracic surgeons so that perioperative risks to the patient is minimized. For one, a retro-aortic innominate vein may be difficult to distinguish and be mistaken for an absent left brachiocephalic vein. Consequentially, the descending portion of the left brachiocephalic vein may be confused for a persistent left superior vena cava, which carries implications for the type of cannulation for systemic venous drainage when performing a cardiopulmonary bypass. Secondly, this structural anomaly can prevent adequate exposure of the surgical field during the construction of a modified Blalock-Taussig shunt or ligation of a patent ductus arteriosus (2). Therefore, early detection of this anomalous innominate vein is important to guide decision-making and improve surgical outcomes. Recent advances in technology have allowed us to carry out pre-natal and post-natal detection of anomalous innominate vein with echocardiography. In the case of diagnostic uncertainty with echocardiography, CT scan can be used to confirm the diagnosis (2). For instance, Choi et al detected 24 cases of a retro-aortic innominate vein out of 2,457 patients (0.98%) with CHD who underwent echocardiogram investigation(8) whereas, Chen et al. reported a higher incidence (1.7%) when using CT scan. Cardiac magnetic resonance imaging (MRI) can also be used for further evaluation if doubts about the diagnosis remain after echocardiography (2, 4), which was not our experience in this patient. Although both cardiac MRI and CT scan can be used to identify the presence of an anomalous innominate vein, CT scan is the preferred choice as it can be performed with mild sedation, while MRI examination in children requires general anesthesia. Additionally, the spatial resolution of CT is better compared to MRI and therefore, provides greater detail when it comes to anatomical analysis of the arterial and venous structures surrounding the heart. On the other hand, MRI scan is more useful for functional evaluations of the heart and detecting intracardiac malformations as well as cardiac wall abnormalities (9). In our experience, accurate pre-operative echocardiographic detection of this vascular anomaly has enhanced planning and decision-making for pediatric cardiologists and cardiac surgeons, without need for a CT or an MRI scan.

## Concluding remarks

In summary, clinicians should be aware of the potential of respiratory complications in patients with a combination of RAA and retro-aortic innominate vein when associated with tetralogy of Fallot. Furthermore, the presence of an anomalous innominate vein may complicate clinical procedures and cardiac surgery for the unsuspecting clinician. Therefore, early detection of these cardiovascular anomalies is crucial to guide management and consequently improve patient outcomes.

## Parent/guardian consent

Written and informed consent was obtained from the parent/guardian of the patient for publication of this case report.

## Author contributions

ZL and BC prepared the entire manuscript. AC proposed the idea for this case report and revised and edited the prepared manuscript. SY provided the images and edited the prepared manuscript.

### Conflict of interest statement

The authors declare that the research was conducted in the absence of any commercial or financial relationships that could be construed as a potential conflict of interest.
